# Aortic Dissection Auxiliary Diagnosis Model and Applied Research Based on Ensemble Learning

**DOI:** 10.3389/fcvm.2021.777757

**Published:** 2021-12-23

**Authors:** Jingmin Luo, Wei Zhang, Shiyang Tan, Lijue Liu, Yongping Bai, Guogang Zhang

**Affiliations:** ^1^Xiangya Hospital of Central South University, Changsha, China; ^2^Information Science and Engineering School of Central South University, Changsha, China; ^3^Third Xiangya Hospital of Central South University, Changsha, China

**Keywords:** aortic dissection, early detection, artificial intelligence, diagnosis model, RS-easy ensemble

## Abstract

Aortic dissection (AD), a dangerous disease threatening to human beings, has a hidden onset and rapid progression and has few effective methods in its early diagnosis. At present, although CT angiography acts as the gold standard on AD diagnosis, it is so expensive and time-consuming that it can hardly offer practical help to patients. Meanwhile, the artificial intelligence technology may provide a cheap but effective approach to building an auxiliary diagnosis model for improving the early AD diagnosis rate by taking advantage of the data of the general conditions of AD patients, such as the data about the basic inspection information. Therefore, this study proposes to hybrid five types of machine learning operators into an integrated diagnosis model, as an auxiliary diagnostic approach, to cooperate with the AD-clinical analysis. To improve the diagnose accuracy, the participating rate of each operator in the proposed model may adjust adaptively according to the result of the data learning. After a set of experimental evaluations, the proposed model, acting as the preliminary AD-discriminant, has reached an accuracy of over 80%, which provides a promising instance for medical colleagues.

## Introduction

Aortic dissection (AD) is a dangerous cardiovascular disease with complex pathogenesis. AD cause damage in the aortic intima and tunica media membrane structure, blood in the aortic lumen flows into the tunica media from the tear of aortic intimal film, causing membrane separation along the aorta extending along the long axis of cavities and false cavities of the aortic wall separating state, and cause the corresponding symptoms like chest pain. Incidences of AD each year are about 5–10 cases per million population and peak ages are 50–70 years old, with the ratio of male to female about 2–3: 1.65–70%, all have a hidden onset and rapid progression (1). AD prognosis is poor without timely treatment, patients shall receive a quick death because of the acute complications such as cardiac tamponade and arrhythmia (2).

At present, acknowledged etiology of AD is relatively limited and known risk factors associated with AD include vascular endothelial damage and high blood pressure (1). Due to the hidden onset and rapid progression of AD in the early phase leading to higher mortality, many primary medical institutions, without appropriate equipment such as CT, have serious difficulty providing early diagnosis and prognosis of AD. Most doctors of these basic level medical institutions diagnose AD based on their experience instead of with CT angiography because tests are expensive and take a long time due to their complexity. Since only a handful of large hospitals are equipped with the necessary equipment, many AD patients who first come to a basic level hospital are not accurately diagnosed and treated, which means they lose the best chance of getting effective treatment or referral (3). Therefore, establishing an early auxiliary diagnosis model of AD recognition that uses routine medical data that can be gained by basic level medical staff is an urgent and important task.

Using an auxiliary diagnosis model based on artificial intelligence is a hot topic in the field of biomedical engineering, and it is a very good example of combining multiple technologies, such as computer science, big data technology, cognitive science, and logic, with the practice of medicine. There has been great progress in using artificial intelligence assisted diagnostic technology in diagnosis of AD, such as using machine learning model to classify aortic dissection patients. Da Huo et al. used Weka toolkit to employ four common classifiers on dataset including Bayesian Network, Naïve Bayes, J48 and SMO, and found Bayes Net model has the best performance among four classifiers (4). Lijue Liu et al. used multiple machine learning models, include AdaBoost, SmoteBagging, EasyEnsemble and CalibratedAdaMEC, to study aortic dissection screening method, and found that the screening performance of the models had a misdiagnosis rate lower than 25% except AdaBoost (5). These research showed that artificial intelligence technology can help clinicians build a new early screening approach for AD.

Although machine learning methods are helpful for the diagnosis of AD, the high death rate of AD requires higher accuracy and single modeling and analysis method is not enough. Ensemble learning strategies have demonstrated impressive capacities to improve the prediction accuracy of base learning algorithms. Zhenya Qi et al. used *t*-test to investigate if the performance of an ensemble for heart disease prediction, which contains five heterogeneous classifiers: random forest, logistic regression, support vector machine, extreme learning machine and k-nearest neighbor, was better than individual classifiers and the contribution of Relief algorithm (6). The best performance was achieved by the proposed method according to 10-fold cross validation. The statistical tests demonstrated that the performance of the proposed ensemble was significantly superior to individual classifiers, and the efficiency of classification was distinctively improved by Relief algorithm (6). Therefore, establishing an auxiliary diagnosis model by using artificial intelligence technology and applying it to the early diagnosis of AD should increase the positive diagnostic rate of AD greatly. At the same time, the establishment of a model may improve the survival rate and prognosis of AD patients.

## Methods

### Case Information Collection

After signing all relevant documents, including the confidentiality agreements and ethical review document, patient‘s information are retrieved from the Xiangya hospital's electronic medical record system and statistics from 2006 to 2016. A total number of 3,249 AD patients' and another 95,711 cases of non-AD patients‘ clinical data had been collected. All these data were divided into three categories: (1) patients' basic information: gender, age, height, weight, family history, past medical history, personal habits; (2) text messages about symptoms and complaints: presence of chest pain, heart palpitations, dizziness or headache symptoms, frequency and extent, presence of abnormal blood vessel pulsing, aortic valve area signs such as noise; (3) quantitative index of tests and examinations: Blood routine, coagulation routine, hepatorenal function, etc.

### Indexes Screening

The indexes chosen for this research come mainly from the following tests and examinations: blood routine (BR), coagulation routine (CR), hepatorenal function and serum lipid, myocardial enzymology, and serum electrolyte. These indexes are commonly used in basic level medical institutions. Furthermore, results can be quickly obtained after sampling, meaning it takes less time to judge the situation of a patient compared to some examinations that require special equipment, such as coronary CTA and CT angiographic. If these indexes, which used to have little significance in AD diagnosis, can actually play an important role in some way, they may be irreplaceable to AD patients because of their cheap price and accessibility.

BR is one of the most commonly used laboratory tests in clinical laboratories and the basic principle is using electrical impedance and spectrophotometric colorimetry to detect if the blood's composition, composed of red blood cells, white blood cells and platelets, is in the normal range. The number and normal of size of cells, can be used to identify if the cells are normal. BR can also figure average density and conversion of various types of cells in the blood by formula, such as hematocrit, mean corpuscular volume, or mean corpuscular hemoglobin. Using BR to assess the patient's blood type and quantity of cells may help detect significant hemodynamic changes in the early phase of AD. Existing studies have shown that 43% of AD patients have different degrees of BR abnormalities (7). Therefore, 22 indexes of BR are taken from Xiangya hospital into further analysis (Table 1).

**Table 1:** Twenty two indexes of blood routine (BR) from Xiangya hospital.

**Index name**	**Unit**	**Reference range**
White blood cells count	10‘9/L	3.5–9.5
Red blood cells count	10‘12/L	3.85–5.1
Hemoglobin	g/L	115–150
Thrombocyte	10‘9/L	125–350
Hematocrit	%	35.0–45.0
Neutrophil granulocyte count	10‘9/L	1.8–6.3
Lymphocyte count	10‘9/L	1.1–3.2
Eosnophils granulocyte count	10‘9/L	0.02–0.52
Basophilic granulocyte count	10‘9/L	0.00–0.6
Monocyte count	10‘9/L	0.1–0.6
Neutrophil percentage	%	40.0–75.0
Lymphocyte percentage	%	20.0–50.0
Eosnophils percentage	%	0.1–1.0
Basophilic percentage	%	0.4–8.0
Monocyte percentage	%	3.0–10.0
Mean corpuscular volume	fl	82.0–100.0
Mean corpuscular hemoglobin	PG	27.0–34.0
Mean corpuscular hemoglobin concentration	g/L	316–354
Red blood cells distribution width	%	<15
Thrombocytocrit	%	0.18–0.22
Mean platelet volume	fl	7.6–13.2
Platelet distribution width	%	<17.2

CR is an activity to detect various coagulation factors with solidification method, hair color substrate method and immune turbidity method by using photoelectric principle. CR can assess a patient's blood coagulation function and bleeding risk from the activity of coagulation factors such as PT, APTT, TT and FBG in the blood. Research shows that a considerable number of preoperative AD patients have lower levels of coagulation factor than normal and the MAp MAf is in a state of relative hyperthyroidism. This may be because the blood contact with the endothelium of the false lumen height of thrombin activation in the early phase of AD. Because acute AD patients' onset time is very short, blood coagulation function is still in the stage of high activation of high condensation, and under the action of thrombin, the expression of platelet fibrinogen are increased. The indexes od CR, D—dimer especially have been proven to have guiding function in the process of AD diagnosis (8). Thus, 10 indexes of CR are taken from Xiangya hospital into further analysis (Table 2).

**Table 2:** Ten indexes of coagulation routine (CR) from Xiangya hospital.

**Index name**	**Unit**	**Reference range**
Prothrombin time (PT)	sec	10.0–16.0
Prothrombin percentage (PP)	%	70–140
International normalized ratio (INR)	–	0.8–1.2
Activated partial thromboplastin time (APTT)	sec	25.0–43.0
Thrombin time	sec	14.0–21.0
Fibrinogen	g/L	2.0–4.0
Plasma fibrinogen degradation products	mg/L	0–5
D - dimer	mg/L	0–0.5
Plasma plasminogen antigen	mg/L	230–386
Plasma antithrombin III antigen	mg/L	180–392

Hepatorenal function and myocardial enzymology are commonly used tests to detect several important organs‘ function, like heart, liver and kidney, and whole body metabolic function in clinical way. These tests mainly use the enzyme circulation method, enzyme coupling method, continuous monitoring method, Reitman colorimetric method and fluorescent method to complete the systematic analysis of indicators related to the metabolism of an organ. The tests can reflect the basic situation of important organs and whole body metabolic function. In the early phase of AD, the effects of hemodynamic changes on metabolism may be subtle, but they do exist. Due to the organizational microenvironment disorder and myocardial damage caused by hemodynamic changes, some abnormal results may be reflected in the results of such tests (9, 10). Thus, 18 indexes of hepatorenal function and myocardial enzymology are taken into further analysis (Table 3).

**Table 3:** Eighteen indexes of hepatorenal function and myocardial enzymology from Xiangya hospital.

**Index name**	**Unit**	**Reference range**
Total protein	g/L	65.0–85.0
Albumin	g/L	40.0–50.0
Globulin	g/L	20.0–40.0
A/G	–	1.2–2.4
Total bilirubin	μ mol/L	1.7–17.1
Direct bilirubin	μ mol/L	0.0~6.8
Total bile acid	μ mol/L	0.0–12.0
Glutamic-pyruvic transaminase	U/L	7.0–40.0
Glutamic oxalacetic transaminase	U/L	13.0–15.0
Glycated serum protein	mmol/L	1.18–2.20
Urea nitrogen	mmol/L	3.10–8.80
Creatinine	mmol/L	41.0–111.0
Trioxypurine	μ mol/L	155.0–357.0
Glucose	μ mol/L	3.90–6.10
Lactic dehydrogenase	U/L	120.0–250.0
Creatine kinase	U/L	40.0–200.0
Creatine kinase isoenzymes	U/L	<24.0
Myohemoglobin	μ g/L	<70.0

Serum lipid is a routine test given to patients with cardiovascular diseases since abnormal lipids metabolism is believed to have an important relationship with angiocardiopathy. The purpose of this test is to analyze blood lipid levels and species of patients through the Cholesterol oxidase-peroxidase-anti-peroxidase method (COD—PAP method) and super centrifugal plasma combined with selective precipitation. As one of the most common risk factors for coronary heart disease, blood lipid results can also be an indirect risk factor for AD patients. Lipid metabolism may allow us to assess AD patients' vascular stability and risk of sudden death by indirect methods (11). Therefore, five indexes of serum lipid are also taken into further analysis (Table 4).

**Table 4:** Five indexes of serum lipid from Xiangya hospital.

**Index name**	**Unit**	**Reference range**
Total triglyceride (TG)	mmol/L	<1.70
Total cholesterol (TC)	mmol/L	<5.18
High-density lipoprotein (HDL)	mmol/L	1.04–1.55
Low-density lipoprotein (LDL)	mmol/L	1.55–3.19
HDL/TC	-	0.17–0.45

Serum electrolyte is the test that detects specific ion concentrations in serum. The concentration of several important ions that maintain balance of osmotic pressure between blood and interstitial fluid, such as sodium, chloride, and calcium, are measured by the ion selective electrode method. It is not surprising to find that hemodynamic changes of AD patients affect their level of serum electrolytes in blood and tissue fluid microenvironment (12, 13). Therefore, 8 indexes of serum electrolyte are taken from Xiangya hospital into further analysis (Table 5).

**Table 5:** Eight indexes of serum lipid from Xiangya hospital.

**Index name**	**Unit**	**Reference range**
Potassium	mmol/L	3.50–5.30
Sodium	mmol/L	137.0–147.0
Chlorine	mmol/L	99.0–110.0
Calcium	mmol/L	2.00–2.60
Phosphorus	mmol/L	0.86–1.78
Magnesium	mmol/L	0.66–1.07
Carbon dioxide (CO_2_)	mmol/L	19.0–33.0
Anion gap (AG)	mmol/L	8.0–16.0

### Modeling Method and Data Mining

Research adopts the following main modeling methods: linear discriminant analysis (LDA), artificial neural networks (ANNs), decision tree (DT), support vector machine (SVM) and EasyEnsemble learning. LDA is one of the most classic analyses to classify statistical models of simple discriminant function. In the feature space, LDA can be used to compare different categories of discriminant function value size and classify them by analyzing different categories (14). ANNs is a method to calculate by simulating the way of people thinking. It is a non-linear dynamic system and has the characteristics of distributed storage and parallel collaborative information processing. Back Propagation (BP) neural network algorithm, which is also known as the error Back Propagation algorithm, and it is an artificial neural network supervised learning algorithm. In theory, the BP algorithm can approximate arbitrary functions because its basic structure is composed of a non-linear change unit and it has a strong non-linear mapping ability. Furthermore, due to parameters such as number of the middle layer, processing units of each layer and network coefficient of learning can be set according to the concrete situation; BP has great flexibility (15). DT is an algorithm to calculate the net present value of the probability of expectation, which is greater than or equal to zero based on the probability of known situations. Using a decision tree, the project risk and feasibility evaluation can be easily done with a graphic method. To be specific, DT is a prediction model with tree structure, and it represents the object properties and a mapping relationship between object values, where each internal node is a test on an attribute; each branch represents a test output; each leaf node represents one kind of category (16). SVM is a kind of supervised learning model related to the relevant learning algorithm and mainly used to analyze data and recognize patterns in classification and regression analysis. The statistical learning theories that SVM is based on, like Vapnik-Chervonenkis (VC) dimension theory and structure risk minimum principle, enable SVM to get the best generalization ability and seek the best compromise between the complexity of the model (i.e., on a particular learning accuracy of training samples) and learning ability (i.e., the ability to identify random sample correctly) according to the sample of limited information (17).

**Figure 1:**
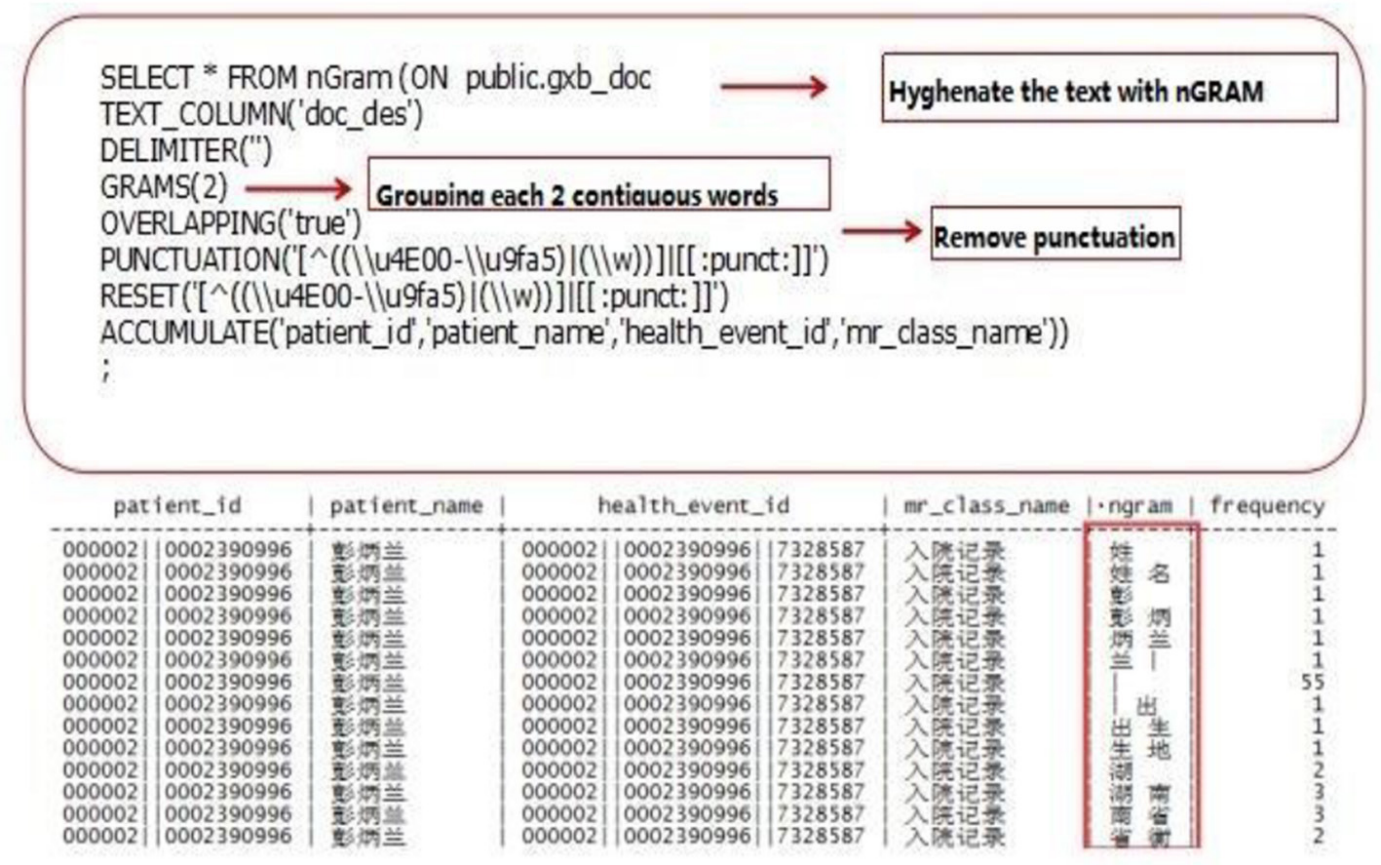
Preliminary discrimination of keywords.

**Figure 2:**
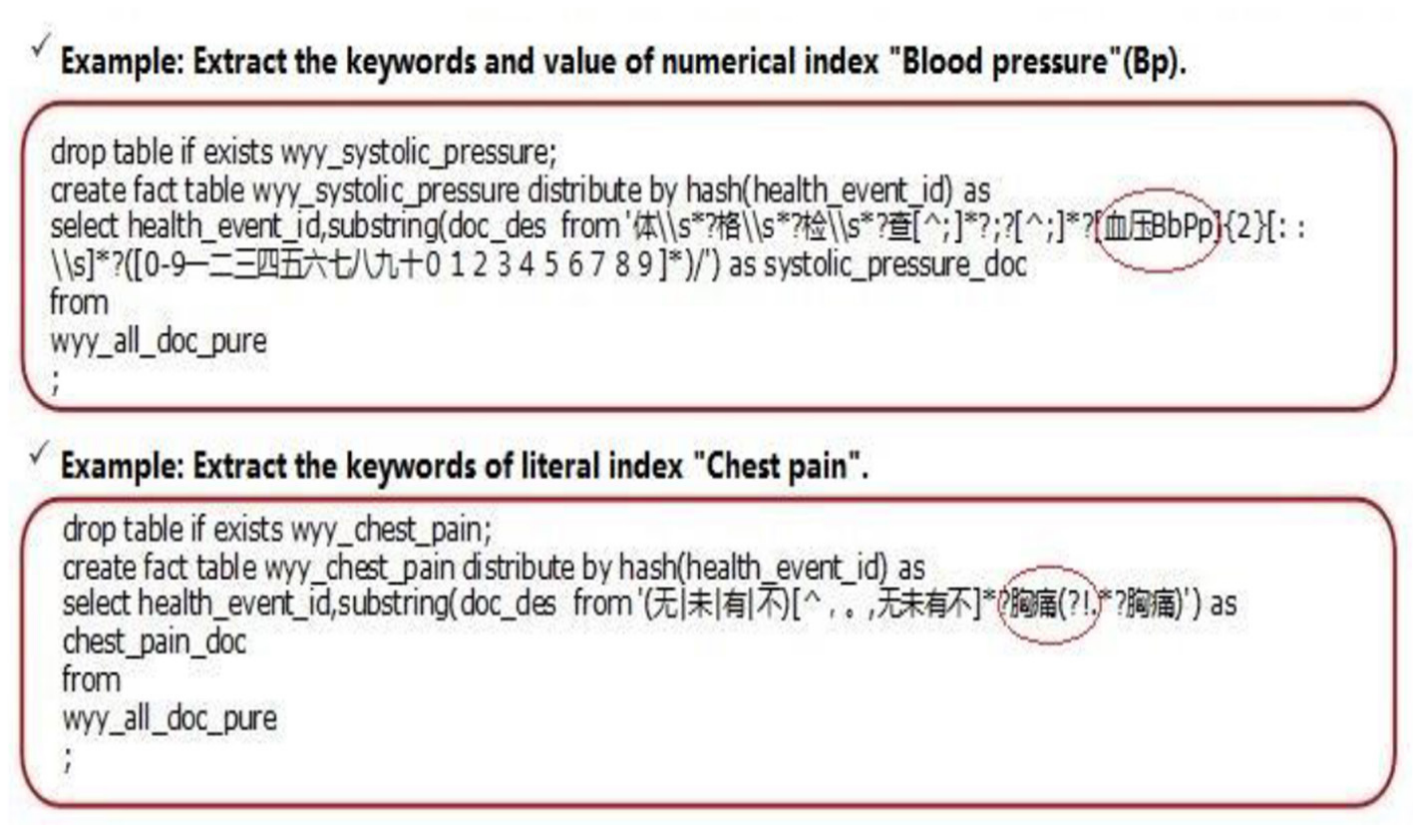
Secondary screening of keywords.

All modeling data mining is given operation with nGram function of a decision tree model and session texts are segmented in pairs and get the keyword combination table (Figure 1). Each word combination contains two words and the high frequency word combinations will be picked out after statistic analysis. Then the words that already exist in the table are filtered out and the possible keywords combinations (Figure 2) are obtained. Finally, there is human intervention for keyword selection and confirming official keyword combinations (Table 6).

**Table 6:** Text indexes of basic information and complaint.

**Indexes**	**Type**	**Value/unit**	**Notes**
**Main complaint**			
Thoracalgia	Text	①Yes ②No	Yes = 1, No = 0 (below)
Stomachache	Text	① Yes ②No	
Palpitation	Text	① Yes ②No	
Dizziness or Headache	Text	① Yes ②No	
Abnormal pulse	Text	① Yes ②No	
Aortic area murmur	Text	① Yes ②No	
**Family history**			
Hypertension	Text	① Yes ②No	
Diabetes	Text	① Yes ②No	
Marfan syndrome	Text	① Yes ②No	
Aortic dissection	Text	① Yes ②No	
**Medical history**			
Chest trauma	Text	① Yes ②No	
Marfan syndrome	Text	① Yes ②No	
Time of Marfan syndrome	Numerical	Year	
Hypertension	Text	① Yes ②No	
Time of Hypertension	Numerical	Year	
Diabetes	Text	① Yes ②No	
Time of Diabetes	Numerical	Year	
**Basic information**			
Age	Numerical	Year	
Gender	Text	①Male ②Female	Male = 1, Female = 0
Heart rate	Numerical	Beats/min	
Systolic pressure	Numerical	mmhg	
Diastolic pressure	Numerical	mmhg	
Smoking status	Text	①Yes ②No③Quit	Yes = 1, N0 = 0, Quit = 2
Time of Smoking	Numerical	Year	
Time of Quitting Smoking	Numerical	Year	
Drinking status	Text	①Yes ②No③Quit	Yes = 1, N0 = 0, Quit = 2
Time of Drinking	Numerical	Year	
Time of Quitting Drinking	Numerical	Year	
Aortic dissection	text	① Yes ②No	Target variable

### Modeling Parameters and the Training Process

**Figure 3:**
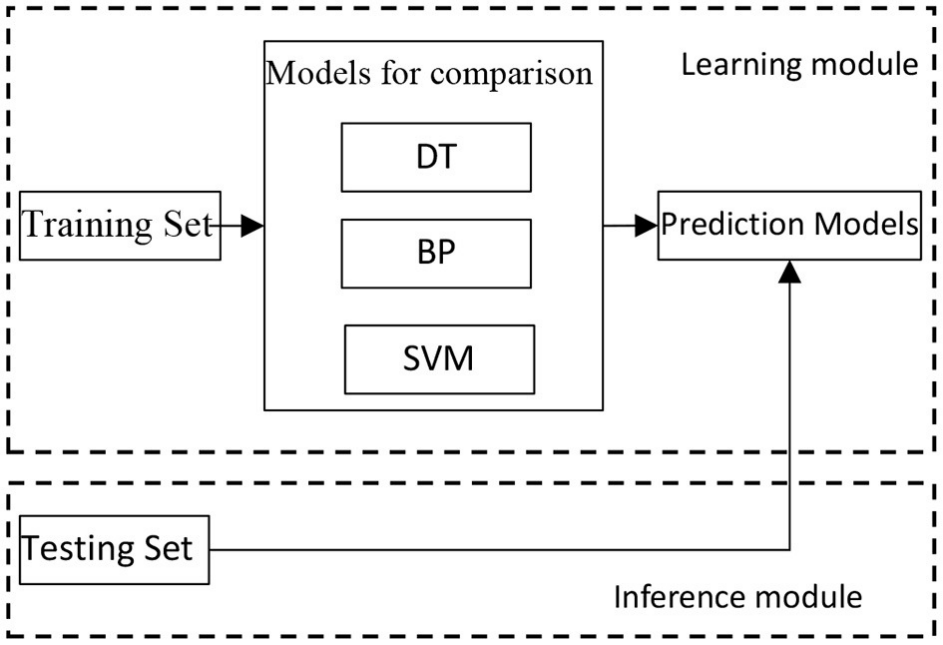
Comparison of modeling method algorithms.

**Figure 4:**
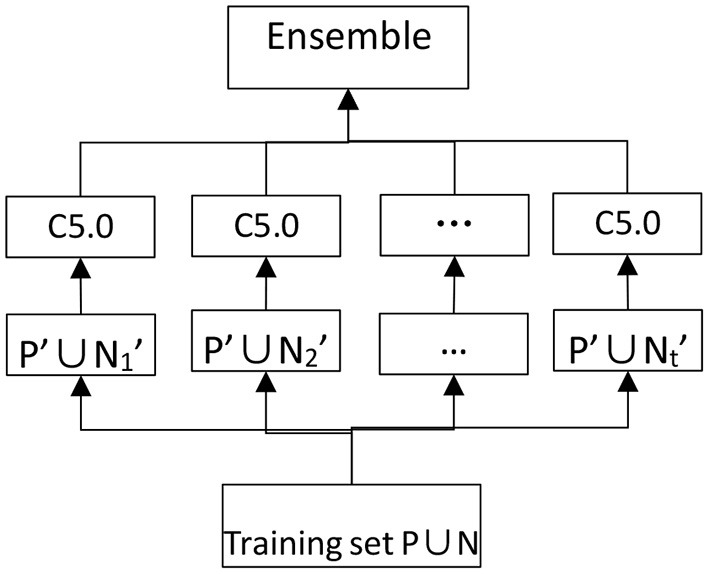
Eliminate imbalances of modeling method.

Because of the uncertainty of the AD on each patients, the majority of data that patients provide to doctors are fuzzy and inaccurate. Inevitably, one must try a variety of modeling methods in the process of creating an AD auxiliary diagnosis aneurysm model. According to analysis of the actual AD diagnosis process and knowledge of artificial intelligence and medical statistics, a variety of methods are built to analyze the data and compare the effect of different models (Figure 3). The goal of this research is not only to gain an auxiliary diagnosis aneurysm model that simulate the diagnosis of AD, but also adaptively adjust diagnosis model parameters and improve the accuracy of the auxiliary diagnosis with the accumulation of cases. After eliminating imbalances of each modeling method, the final ensemble learning model is established (Figure 4). All clinic data are organized by the Excel files and numbering (Supplementary Materials 1, 2). Finally 80 features are used and obtained corresponding data from the electronic medical record and number all features as 1–80. These features were input into the machine learning model as feature vectors.

#### Linear Discriminant Analysis Model

For the *i*th feature vector *X*_*i*_= (*x*__*i*_1_, *x*__*i*_2_ ..., *x*__*i*_80_), LDA calculate the discriminant functions *g*_*c*_(*X*_*i*_)=$X_{i}W_{c}^{\text{T}}$, where *c* ∈{1, 2} is the number of categories, *X*_*i*_ is the feature vector of the *i*th sample, *W*_*c*_=(*W*__*c*_1_ *W*__*c*_2_..., *W*__*c*_80_) is the weight vector of class *c*. The final decision result depends on which category of decision function has a larger value.

#### BP Neural Network Model

BP neural network is an ANNs algorithm and its basic idea is a kind of gradient descent method. BP creates the minimum actual output value of the network and the minimum desired output error of the mean square error by using gradient search technology. The function of one neuron is to seek the inner product of input vectors and weight vectors, and obtain a scalar result via a non-linear transfer function. The individual neurons split an n-dimensional vector space into two parts with a hyperplane and make a judgment that the vector belong to which side of the hyperplane by given an input vector. The BP network applied in these paper was a 2 layer network with 80 input unit, 1 hidden layer and 1 output unit.

#### Decision Tree Model

DT establishes the model of decision-making based on data attribute tree structure, and it is often used to solve classification and regression problems. Common algorithms of DT include Classification and Regression Tree (CART), ID3, C4.5, and Random Forests (RF). DT classification can be used as a prediction model and it represents a mapping between object and object attribute values. From the root node, each minor node can express attributes of object judgment conditions, while the branch is representative of the object that meets the requirements and the leaves nodes are the object's prediction results.

#### Support Vector Machine Model

SVM modeling depends on different Kernel functions, such as the kernel function including Linear Kernel (LK), Polynomial Kernel (PK) and Radial Basis Kernel Function (RBF). RBF is also known as the Gaussian Kernel (GK), The operation results of SVM default parameters are satisfactory, so some adjustments are made in the penalty coefficient and kernel functions. The final parameters are penalty coefficient C = 0.125; kernel = “rbf;” degree = 3; gamma = 0.0078125.

#### EasyEnsemble Model

EasyEnsemble is a kind of integrated learning which deals with big categories of data by using the down-sampling method with Adaboost as a weak classifier, and it is an efficient path to dealing with unbalanced data. Data ratio is severely unbalanced due to the rarity of AD; the amount of data from AD patients is very small compared with non-AD patients, and the two kinds of data have an ~1: 65 ratio imbalance. Therefore, EasyEnsemble is the best choice to build an effective model. The specific practices are as follows: Assuming P as the dataset of the minority class, and N as the dataset of the majority class, |P| and |N| as the cardinal number meet the condition that |N| > > |P|. The dataset of the majority class is divided into several subdatasets like class N1, N2 and N3...NT, for any dataset Ni (1 < I < T), give | Ni | = | P|. Each Ni dataset will be trained to be a classifier Hi by combining with P as training set. AdaBoost is used to train Hi in this algorithm and make up the final model through the combination of classifier with number T.

## Results

### Data Preliminary Processing and Screening

All relevant data are exported from the electronic medical records of Xiangya hospital and obtained a total of 3,249 AD patients' medical records and 95,711 cases of non-AD patients' medical records from 2006 to 2016. After preliminary statistical analysis and exclusion of patients with undetermined diagnoses or incomplete data, a total of 53,213 cases are taken as the modeling cases, including 802 AD patients and 52,411 non-AD patients and finally 43 indexes, which *P* ≤ 0.001, are designated as model analysis indexes (Table 7).

**Table 7:** Forty-three indexes of modeling analysis.

**Indexes**	**AD patients (*N* = 802)**	**Non-AD patients (*N* = 52,411)**	**t/Chi**	***p***
Age (Mean ± SD)	55.57 ± 12.90	62.56 ± 13.06	15.03	<0.001
Gender (Male, %)	574 (71.57)	29,994 (57.23)	66.47	<0.001
Thoracalgia	206 (25.79)	9,460 (18.05)	30.99	<0.001
Palpitation	63 (7.86)	6,106 (11.65)	11.10	0.001
Dizziness or headache	62 (7.73)	7,803 (14.89)	32.13	<0.001
Aortic area murmur	23 (2.87)	377 (0.72)	48.88	<0.001
Chest trauma	11 (1.37)	206 (0.39)	18.62	<0.001
Smoking status			85.79	<0.001
Never smoking	296 (36.91)	12,293 (23.46)		
Smoking	485 (60.47)	37,121 (70.83)		
Quit smoking	21 (2.62)	2,997 (5.71)		
Time of smoking	10.22 ± 14.39	7.34 ± 13.88	−5.63	<0.001
Hypertension	530 (66.08)	31,571 (60.24)	11.29	0.001
Diabetes	88 (10.97)	11,910 (22.72)	62.47	<0.001
Time of diabetes	0.85 ± 2.87	1.82 ± 3.83	9.40	<0.001
Heart rate	81.74 ± 13.87	78.73 ± 14.20	−6.10	<0.001
Systolic pressure	142.41 ± 26.71	136.86 ± 21.90	−5.85	<0.001
Diastolic pressure	83.20 ± 16.59	80.46 ± 13.01	−4.66	<0.001
Neutrophil granulocyte count	7.16 ± 4.08	4.79 ± 3.47	−16.35	<0.001
Neutrophil percentage	72.83 ± 10.79	65.30 ± 12.09	−19.59	<0.001
Lymphocyte percentage	16.94 ± 9.10	24.22 ± 10.44	22.43	<0.001
Lymphocyte count	1.36 ± 0.60	1.57 ± 2.03	9.07	<0.001
Mean platelet volume	8.93 ± 1.39	9.36 ± 1.58	8.60	<0.001
Total protein	64.58 ± 7.06	65.43 ± 8.04	3.41	0.001
Albumin	37.08 ± 5.67	38.61 ± 6.26	7.60	<0.001
Globulin	27.57 ± 5.19	26.94 ± 5.32	−3.39	0.001
A/G	1.40 ± 0.36	1.49 ± 0.37	6.77	<0.001
Total bilirubin	16.19 ± 21.62	13.20 ± 26.81	−3.86	<0.001
Glutamic-pyruvic transaminase	66.50 ± 296.27	32.47 ± 108.73	−3.25	0.001
Glycated serum protein	2.25 ± 0.62	2.03 ± 0.73	−9.79	<0.001
Lactic dehydrogenase	322.03 ± 684.10	236.51 ± 283.48	−3.54	<0.001
Myohemoglobin	72.69 ± 84.95	57.60 ± 59.02	−5.01	<0.001
Potassium	3.83 ± 0.56	3.97 ± 0.52	7.52	<0.001
Sodium	139.37 ± 4.28	140.71 ± 3.79	8.77	<0.001
Chlorine	101.08 ± 4.95	102.59 ± 4.62	8.56	<0.001
Calcium	2.16 ± 0.16	2.21 ± 0.18	8.88	<0.001
PP	99.83 ± 18.59	106.62 ± 17.36	10.28	<0.001
INR	1.06 ± 0.39	1.01 ± 0.28	−3.91	<0.001
APTT	37.66 ± 11.29	35.54 ± 9.68	−5.29	<0.001
Fibrinogen	4.44 ± 1.81	3.77 ± 1.22	−10.45	<0.001
D – dimer	1.37 ± 1.94	0.97 ± 1.27	−5.49	<0.001
Plasma plasminogen antigen	252.01 ± 24.57	255.86 ± 27.68	4.40	<0.001
PT	13.57 ± 4.39	13.02 ± 3.06	−3.58	<0.001

### Classification of Model Operation Results

Since thoracalgia is the most important symptom in diagnosing AD, and because it is very difficult to distinguish AD patients with thoracalgia from other patients with ailments such as hypertension or CAD in time, collected research data are divided into three parts: (1) AD patients confirmed by coronary CTA or observed in surgical, (2) non-AD patients with thoracalgia confirmed by coronary CTA or coronary angiogram, (3) non-AD patients without thoracalgia confirmed by coronary CTA or coronary angiogram. Model are built with five methods according to the data sets, namely LDA, BP, DT, SVM and EasyEnsemble. First, all data are divided into four categories: True Positive (TP): predicting outcome and actual outcome are both AD; True Negative (TN): predicting outcome and actual outcome are both non-AD; False Positive (FP): predicting outcome is AD but actual outcome is non-AD; False Negative (FN): predicting outcome is non-AD but actual outcome is AD. The main evaluation indexes are used to assess the model are as follows.

#### Accuracy

Accuracy (A) is the correct identification of all kinds of patients, and it is one of the most important indexes that assesses model prediction efficiency in this research. Specifically, the proportion of each model diagnosing correct real results of the total number of AD patients and non-AD patients are taken as the accuracy of the model. The formula is:


(1) $$\begin{array}{r} A=\frac{TP+TN}{TP+TN+FP+FN}*100\% \end{array}$$

#### Error Rate

Error rate (ER) is the index that represents how many non-AD patients are mispredicted as AD patients. Because of the importance of thoracalgia in AD diagnosis, ER of non-AD patients with thoracalgia and non-AD patients without thoracalgia are calculated separately. The non-AD patients with thoracalgia are expressed as TN_t_ or FP_t_, the non-AD patients without thoracalgia are expressed as TN_nt_ or FP_nt_, Similarly there are ER_t_ and ER_nt_. The formulas:


(2) $$\begin{array}{r} ER_{t}=\frac{FP_{t}}{TN_{t}+FP_{t}}*100\% \end{array}$$

(3) $$\begin{array}{r} ER_{nt}=\frac{FP_{nt}}{TN_{nt}+FP_{nt}}*100\% \end{array}$$

#### Recall

Recall® can tell how many positive examples are predicted correctly according to the original sample. Due to the low incidence of AD, A and ER are not precise enough to assess the model prediction efficiency, so Recall are added to ensure model soundness. The proportion of AD patients that are correctly predicted by each model out of the total number of AD patients is taken as the recall. The formula is:


(4) $$\begin{array}{r} R=\frac{TP}{TP+FN}*100\% \end{array}$$

### Comparison of Prediction Results

The final results of the five models have obvious differences (Table 8). Based on the results, EasyEnsemble is chosen as the main modeling method for the AD auxiliary diagnosis model. It is not the model with the highest accuracy or lowest ER, but its highest recall enables it to discover the most AD patients. After some adjustments and verified model stability through multiple tests, the official algorithm code are finalized. Under the premise of maintaining the EasyEnsemble basic algorithm, number M EasyEnsemble model is constructed for Hi (1 < = I < = M) and took half training characteristics, which were randomly selected from the whole data set, as the new training characteristics of the training set. The final model was acquired when the simple average of number M/2 selected from number M EasyEnsemble complete training finished. The upgrade algorithm called RS - EasyEnsemble and pseudo code of the algorithm are shown. The final model has basically achieved expected goals, and it is ready to put into formal application in clinical work after software development work.

**Table 8:** Prediction efficiency results of modeling five algorithms.

**Model**	**LDA**	**BP**	**DT**	**SVM**	**RS-EasyEnsemble**
Accuracy	78.40%	77.60%	71.79%	83.20%	80.09%
Recall	4.35%	34.78%	20.48%	73.91%	81.11%
Error rate (t)	4.00%	16.00%	0%	20.00%	19.20%
Error rate (nt)	5.77%	9.62%	0%	9.62%	11.80%

RS-EasyEnsemble algorithm code

1. Load in *t* subsets of set *N*, for each subset *T*_*i*_, |*T*_*i*_|=|*P*|, *T*_*i*_ ∩*T*_*j*_ = Φ(*i*≠*j*), let *TS*_*i*_= *T*_*i*_⋃*P, TS*_*i*_ is the training set of the *i*th base classifier of ensemble model, *P* is the set of minority class, *N* is the set of majority class, where |*P*| < < |*N*|. Initial the number of base classifiers M.

2. i=1

3. while i < =M repeat

3.1: Randomly sample some features to create a subspace *S*_*i*_.
3.2 Map the samples to feature subspace *S*_*i*_ to create a new sample set *P*' and *N*', where *P*' and *N*' are the set *P* and *N* in feature subspace *S*_*i*_.
3.3 Using each *T*_*i*_ to train the base classifier *H*_*i*_.
3.4 i=i+1 3: Select a half of best model from the *M* models.

4: Output an Ensemble model *H*(*x*)$H\left(x\right)=Round\left(\frac{2}{M}\overset{\frac{M}{2}}{\underset{i=1}{\sum }}H_{i}\right)$

## Discussion

From the trend of medical science development, the combination of artificial intelligence technology and clinical medical analysis is increasingly tight and plays an important role in early warning and auxiliary diagnosis of many diseases (18, 19). Models built by various algorithms can replicate the process of diagnosing diseases which used to depend on the subjective judgment of a clinician with objective data and can provide a better basis for decision-making by clinical doctors by collecting and analyzing new disease information to update the diagnosis methods. As there is uncertainty in the diagnosis of AD based on medical records that are collected in a hurry, using the auxiliary diagnosis model to help in the diagnosis is a good choice. This research has searched for the appropriate algorithm to build an AD auxiliary diagnosis model with the highest accuracy by trying a variety of algorithms.

LDA is a classical method used to figure out a vector a which make the new ubspace that has both the largest and minimum class distance in same time and construct the prediction model (20). Accuracy of LDA was 78.4% and ERs of thoracalgia and non-thoracalgia patients were 4 and 5.77%. This could indicate the LDA model is overall good and rarely misdiagnoses non-AD patients as AD patients. However, the recall was only 4.35% and this did not meet the level to identify as many AD patients as possible. So LDA was not taken into the final model's building.

The BP neural network basic algorithm includes signal forward-propagating and error back-propagation in two parts (21). The direction of calculation error output is from input to output and changes to output to input when weight and threshold values are adjusted. The accuracy of the BP model was 77.6%, which is lower than the LDA model, and two ERs were higher than the LDA model, too. Though accuracy and ERs of the BP model were not as good as the LDA model, it had better recall with 34.78%. This may not be good enough to be used in practical applications, but the BP model can be consider as a more advantageous than the LDA model in identifying AD patients.

The main method of the DT model is random forests, and it is a classifier that contains multiple decision trees; the output category is decided by the mode of the individual tree output category. Samples can be trained and provide better prediction by using multiple decision trees (22). Two of DT model ERs were reduced to 0%, which means the DT model can identify all non-AD patients. However, the DT model is still not the first choice since 20.48% of recall and 71.79% of accuracy did not enable us to find enough AD patients.

SVM has the advantages of dimension reduction ability, small sample training, and quick sort. The SVM model aims to find a hyperplane as demarcation of two types of training sample segmentation so that it could ensure minimum classification ERs. Furthermore, the linear inseparable samples are raised from a low dimension feature space to higher one so it became linear separable by using kernel function RBF (23). Under the premise of maintaining accuracy at 83.2%, the recall of the SVM model rose to 73.91% from 8.7% in default parameters. Although the ERs of thoracalgia and non-thoracalgia patients were up to 20 and 9.62%, respectively, the SVM model was really close to experimental objective.

RS-EasyEnsemble is the improved method based on EasyEnsemble, and its main principle is random subspace (RS). Since the key of integrated learning is diversity of base classifiers integration, decision forests are made with RS to increase diversity. Specifically, different parts of characteristics are randomly selected when constructing each decision tree, then all of the map samples are putted into a feature subspace and built new decision trees by using samples after mapping (24). Random feature subspace enables the model to effectively avoid dimension disaster or reduce the redundancy feature space when it meet the dimension disaster (25). The random proportion of the feature space is set to 0.5 because it is close to the optimal combination of precision after experiments on several data sets. RS-EasyEnsemble had the highest recall at 81.11%, which means it is the best model to identify AD patients. Though accuracy of 80.09% is a little lower than the SVM model and two ERs of 19.2 and 11.8%, are not ideal, RS-EasyEnsemble is still the last choice of the main modeling algorithm since the goal of this research is to build an auxiliary diagnostic model that discovers the maximum number of AD patients in the early stage, and it is acceptable to have a few more false AD patients in order to reduce AD mortality caused by missing the diagnosis. From this point of view, this AD auxiliary diagnosis model based on RS-EasyEnsemble achieves experimental objective.

According to the analysis of the actual diagnosis process of AD, an auxiliary diagnosis model had been built whose core algorithm is an integrated learning algorithm and tested the prediction efficiency of the model. From the results, in which the accuracy and recall were both over 80%, this auxiliary diagnosis model is proven to do well in simulating AD diagnosis, and it can improve the diagnosis accuracy with the adaptively adjusted diagnosis model parameters with new data. Though there were some disadvantages in this research, such as positive sample sizes being too small, partial information collection not being complete, and raw data text messages being incomplete digital transformation. This AD auxiliary diagnosis model is a positive attempt in solving clinical problems by using artificial intelligence technology and opening some new research directions and ideas in AD diagnosis. After further improvement and software development work, the AD auxiliary diagnosis model may provide good help for basic level medical staff and save more lives of AD patients.

## Data Availability Statement

The raw data supporting the conclusions of this article will be made available by the authors, without undue reservation.

## Ethics Statement

Written informed consent was obtained from the individual(s) for the publication of any potentially identifiable images or data included in this article.

## Author Contributions

JL: complete clinical data collection, data analysis, diagnosis model testing improvement, and article writing. WZ: complete clinical data analysis and diagnosis model building. ST: complete diagnosis model building and testing. GZ: guide clinical data collection. LL: guide diagnosis model building and testing. YB: guide data analysis and article writing. All authors contributed to the article and approved the submitted version.

## Conflict of Interest

The authors declare that the research was conducted in the absence of any commercial or financial relationships that could be construed as a potential conflict of interest.

## Publisher's Note

All claims expressed in this article are solely those of the authors and do not necessarily represent those of their affiliated organizations, or those of the publisher, the editors and the reviewers. Any product that may be evaluated in this article, or claim that may be made by its manufacturer, is not guaranteed or endorsed by the publisher.
