# Supplementary material for: Aortic Dissection Auxiliary Diagnosis Model and Applied Research Based on Ensemble Learning
Source: Front Cardiovasc Med. 2021 Dec 23;8:777757. doi: 10.3389/fcvm.2021.777757 (PMC8733407; doi:10.3389/fcvm.2021.777757)
Supplement: Supplementary file 1 [file Table_1.docx]

| Numerical index | | | Literal index |
| --- | --- | --- | --- |
| Blood routine examination | Biochemical examination | Others | Symptoms and history |
| 1. WBC  2. RBC  3. HGB  4. HCT  5. MCV  6. MCH  7. MCHC  8. PLT  9. NEUT  10. MONO  11. EO  12. BASO  13. LYMPH  14. LYMPH%  15. MONO%  16. NEUT%  17. EO%  18. BASO%  19. RDW  20. PCT  21. MPV  22. PDW   1. PT   24. APTT  25. TT  26. PT%  27. D-Dimer  28. INR  29. FIB | 30.TP  31. ALB  32. GLO  33. GLU  34. BUN  35. UA  36. CRE  37. TBIL  39. DBIL  40. CO2CP  41. Ca  42. P  43. K  44. Na  45. Cl  46. Mg  47. CHO  48. TG  49. HDL  50. LDL  51. CK  52. LDH  53. CKMB  54. MB  55. HBA1C  56. AG  57. ALP  58. TBA  59. CTNI  60. TNTSH  61. ESR  62. ALT  63. AST  64. PCT | 65. Age  66. Heart rate  67. Diastolic pressure  68. Systolic pressure  69. Smoking time | 1. Chest pain 2. Stomach ache   72. Aortic valve area murmur  73. Dizziness and headache  74. Hypertension  75.Family history of hypertension  76.Family history of aortic dissection  77. Chest trauma history  78. Smoking and duration  79. Heart disease  80. Family history of heart disease |

supplementary materials.1 Clinic data numbering
